# Supplementary material for: The Evolution and Characterization of the RNA Interference Pathways in Lophotrochozoa
Source: Genome Biol Evol. 2024 May 7;16(5):evae098. doi: 10.1093/gbe/evae098 (PMC11114477; doi:10.1093/gbe/evae098)
Supplement: evae098_Supplementary_Data [file evae098_supplementary_data.zip › Supplementary_materials.zip!/selected_miRNAs/NoGe_selected_miRNAs/mirdeep_pdf_files/NMRB01001162.1_Notospermus_geniculatus_strain_Ushimado_scaffold1162,_whole_genome_shotgun_sequence_25710.pdf]

Provisional ID : NMRB01001162.1_Notospermus_geniculatus_strain_Ushimado_scaffold1162,_whole_genome_shotgun_sequence_25710
Score total : 106804
Score for star read(s) : 3.9
Score for read counts : 106795.2
Score for mfe : 1.8
Score for randfold : 0
Score for cons. seed : 3
Total read count : 209486
Mature read count : 209327
Loop read count : 0
Star read count : 159

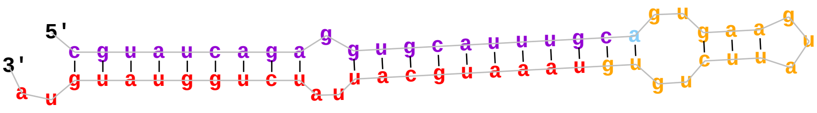

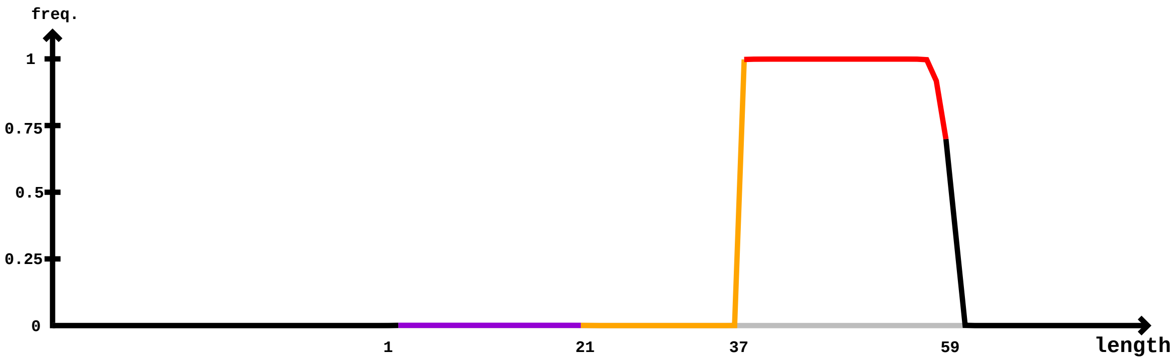

Star Mature

5'- cuagauguuguuuuguugauaccuguggaggguucguaucagaggugcauuugcagugaaguauucuguguaaaugcauuaucugguauguaacucucgcauguugugcagc -3'	obs		
cuagauguuguuuuguugauaccuguggaggguucguaucagaggugcauuugcagugaaguauucuguguaaaugcauuaucugguauguaacucucgcauguugugcagc	exp		
.............((((.(((.((((((((((((((((((((.(((((((((((..(((...)))..)))))))))))..))))))))).))))))).))))..)))))))	reads	mm	sample
..................................cguaucagaggugcauuugc..	26	0	Mm1
..................................cguaucagaggugcauuugca...	16	0	Mm1
..................................cguaucagaggugcauuAgca...	1	1	Mm1
....Cuaaaugcauuaucugguaugua....................	1	1	Mm1
....uaaaugcauuaucuggua........................	4	0	Mm1
....uaaaugcauuaucugguau.......................	64	0	Mm1
....uaaaugcauuaucugguUug......................	4	1	Mm1
....uaaaugcauuaucugguGug......................	4	1	Mm1
....uaaaugcauuGucugguaug......................	1	1	Mm1
....uaaaugcauuaucugguCug......................	6	1	Mm1
....uaaaugcauuaucugguauU......................	2	1	Mm1
....uCaaugcauuaucugguaug......................	1	1	Mm1
....uaaaugcauuCucugguaug......................	3	1	Mm1
....uaaaugcCuuaucugguaug......................	1	1	Mm1
....uaaaugcauuaucuggGaug......................	2	1	Mm1
....uaaaugcauuaucugguauA......................	1	1	Mm1
....uaaaugcauuaCcugguaug......................	1	1	Mm1
....uaaaugcauuUucugguaug......................	1	1	Mm1
....uaaaugcauuaucugguaug......................	854	0	Mm1
....uaaaugcauuaucuggAaug......................	1	1	Mm1
....uUaaugcauuaucugguaug......................	2	1	Mm1
....uaaaugcauuaucuggCaug......................	5	1	Mm1
....uaaaCgcauuaucugguaugu.....................	2	1	Mm1
....uaaauAcauuaucugguaugu.....................	1	1	Mm1
....uaaaugcauuaucugguaCgu.....................	1	1	Mm1
....uaGaugcauuaucugguaugu.....................	4	1	Mm1
....uaaaugcauuaucugguCugu.....................	33	1	Mm1
....uaaaugcaGuaucugguaugu.....................	1	1	Mm1
....uaaaugcauuGucugguaugu.....................	11	1	Mm1
....uaaauUcauuaucugguaugu.....................	3	1	Mm1
....uUaaugcauuaucugguaugu.....................	4	1	Mm1
....uaaaugcauuUucugguaugu.....................	9	1	Mm1
....uaaaugcaAuaucugguaugu.....................	1	1	Mm1

```
                                   Star                                Mature
cuagauguuguuuuguugauaccuguggaggguucguaucagaggugcauuugcagugaaguauucuguguaaaugcauuaucugguauguaacucucgcauguugugcagc
......................................................................uaaaugcauuaucAgguaugu.....................    3      1    Mm1
......................................................................uaaaugNauuaucugguaugu.....................    2      1    Mm1
......................................................................uaaaugUauuaucugguaugu.....................    3      1    Mm1
......................................................................uaaaugcauuaucugguUugu.....................    24     1    Mm1
......................................................................uaaaugcauuaucuggAaugu.....................    15     1    Mm1
......................................................................uaaaugcauuaucugguaugG.....................    12     1    Mm1
......................................................................uaaaugcauuaucugguGugu.....................    14     1    Mm1
......................................................................uaaaugcauuaucugguaGgu.....................    1      1    Mm1
......................................................................uaaaugcUuuaucugguaugu.....................    1      1    Mm1
......................................................................uaCaugcauuaucugguaugu.....................    1      1    Mm1
......................................................................uaUaugcauuaucugguaugu.....................    1      1    Mm1
......................................................................uaaaugcauuaucuggCaugu.....................    38     1    Mm1
......................................................................uaaaugcauuCucugguaugu.....................    7      1    Mm1
......................................................................uaaaugcGuuaucugguaugu.....................    4      1    Mm1
......................................................................Aaaaugcauuaucugguaugu.....................    1      1    Mm1
......................................................................Gaaaugcauuaucugguaugu.....................    1      1    Mm1
......................................................................uaaaugcauuaucuggGaugu.....................    18     1    Mm1
......................................................................uaaaugcauuaucGgguaugu.....................    2      1    Mm1
......................................................................uaaaAgcauuaucugguaugu.....................    1      1    Mm1
......................................................................uaaaugcauuaucugguaugu.....................    7427   0    Mm1
......................................................................uaaaugcauuaucugguauCu.....................    1      1    Mm1
......................................................................uaaGugcauuaucugguaugu.....................    1      1    Mm1
......................................................................uGaaugcauuaucugguaugu.....................    5      1    Mm1
......................................................................uaaaugcauuaucugAuaugu.....................    1      1    Mm1
......................................................................Naaaugcauuaucugguaugu.....................    4      1    Mm1
......................................................................uaaaugcauuaCcugguaugu.....................    1      1    Mm1
......................................................................uaaaugcauGaucugguaugu.....................    1      1    Mm1
......................................................................uCaaugcauuaucugguaugu.....................    1      1    Mm1
......................................................................Aaaaugcauuaucugguaugua....................    1      1    Mm1
......................................................................uaaaugcauuaucugguauguU....................    171    1    Mm1
......................................................................uaaGugcauuaucugguaugua....................    1      1    Mm1
......................................................................uaaaugcauuaucCgguaugua....................    3      1    Mm1
......................................................................uaaaugUauuaucugguaugua....................    1      1    Mm1
......................................................................uaaaugcauuUucugguaugua....................    9      1    Mm1
......................................................................uaaaugcauuauUugguaugua....................    1      1    Mm1
......................................................................uaaaugcauuaucAgguaugua....................    1      1    Mm1
......................................................................Caaaugcauuaucugguaugua....................    1      1    Mm1
......................................................................uaGaugcauuaucugguaugua....................    5      1    Mm1
......................................................................uaaauAcauuaucugguaugua....................    1      1    Mm1
......................................................................uaaaugcauuaucugguauguG....................    5      1    Mm1
......................................................................uaaaugcauuaucugguGugua....................    25     1    Mm1
......................................................................uaaauUcauuaucugguaugua....................    1      1    Mm1
......................................................................uaaauCcauuaucugguaugua....................    1      1    Mm1
......................................................................uaUaugcauuaucugguaugua....................    1      1    Mm1
......................................................................uaaaugcaGuaucugguaugua....................    1      1    Mm1
......................................................................uaaaugcauuaucuggGaugua....................    18     1    Mm1
......................................................................uaaaugcauuGucugguaugua....................    9      1    Mm1
......................................................................uaaaugcauuaucugguaugua....................    6809   0    Mm1
......................................................................Naaaugcauuaucugguaugua....................    8      1    Mm1
......................................................................uaaaugcGuuaucugguaugua....................    3      1    Mm1
......................................................................uaaaugcauuaucuggAaugua....................    14     1    Mm1
......................................................................uaaaugcauuaAcugguaugua....................    1      1    Mm1
......................................................................uaaaugcauuaucugguauAua....................    1      1    Mm1
......................................................................uaaaugcauGaucugguaugua....................    1      1    Mm1
......................................................................uaaaugcauuaucugguauUua....................    1      1    Mm1
......................................................................uaaaugcauuaucuggCaugua....................    28     1    Mm1
......................................................................uaaaugcauuaucugguUugua....................    13     1    Mm1
......................................................................uaaaugcauuaucugguaugCa....................    1      1    Mm1
......................................................................uaaaugcauCaucugguaugua....................    1      1    Mm1
......................................................................uaaaugcauuaucugguCugua....................    27     1    Mm1
......................................................................uaaaugcauuaucugguaugGa....................    1      1    Mm1
......................................................................uaaaugcUuuaucugguaugua....................    3      1    Mm1
......................................................................uGaaugcauuaucugguaugua....................    4      1    Mm1
......................................................................uaaaugcauuaucGgguaugua....................    1      1    Mm1
......................................................................uaaaugcauuaucugUuaugua....................    2      1    Mm1
......................................................................uaaaugcauuCucugguaugua....................    5      1    Mm1
......................................................................uUaaugcauuaucugguaugua....................    5      1    Mm1
......................................................................uaaaugcGuuaucugguauguaa...................    1      1    Mm1
......................................................................uaaaugcauuaucugguGuguaa...................    2      1    Mm1
......................................................................uaaaugcauuaucugguauguaG...................    29     1    Mm1
```

Star **Mature**

cuagauguuguuuuguugauaccuguggaggguucguaucagaggugcauuugcagugaaguauucugugauaaaugcauuaucugguauguaacucucgcauguugugcagc			
....uaaaugcauuaucugguauguaU...................	72	1	Mm1
....uaaaugcauuCucugguauguaa...................	2	1	Mm1
....uaaaAgcauuaucugguauguaa...................	1	1	Mm1
....uaaaugcauuaucuggCauguaa...................	3	1	Mm1
....uaaaugcauuaucugguauguaa...................	830	0	Mm1
....uaaaugcauuaucuggGauguaa...................	2	1	Mm1
....uaaaugcauuaucugguUuguaa...................	5	1	Mm1
....uaaaugcauuaucugguCuguaa...................	6	1	Mm1
....uaaaugcauuaucugguauguaac..................	3	0	Mm1
....uaaaugcauuaucugguauguaaU..................	7	1	Mm1
....uaaaugcauuaucugguauguaaA..................	15	1	Mm1
....aaaugcauuaucugguaug......................	1	0	Mm1
....Uaaugcauuaucugguaugu.....................	1	1	Mm1
....Uaaugcauuaucugguaugua....................	1	1	Mm1
....aaaugcauuaucugguaugua....................	6	0	Mm1
....aaaugcauCaucugguaugua....................	1	1	Mm1
....gcauuaucugguauguaG...................	1	1	Mm1
....gcauuaucugguauguaU...................	6	1	Mm1
..................................cguaucagaggugcauuugU..	1	1	Mm2
..................................cguaucagaggugcauuugca...	3	0	Mm2
....Cuaaaugcauuaucugguaug......................	1	1	Mm2
....uaaaugcauuaucuggua........................	1	0	Mm2
....uaaaugcauuaucugguau.......................	16	0	Mm2
....uaaaugcauuaucugguaug......................	418	0	Mm2
....uaaaugcauuaucugguGug......................	1	1	Mm2
....uaaaugcauuCucugguaug......................	1	1	Mm2
....uaaaugcauuGucugguaug......................	3	1	Mm2
....uaaaugcauuaucugguCug......................	2	1	Mm2
....uaaaugcauuaucugguUug......................	1	1	Mm2
....uaaaugcauuaucuggCaug......................	1	1	Mm2
....uaaaugcauuaucuggAaug......................	1	1	Mm2
....uaaaugcauGaucugguaugu.....................	1	1	Mm2
....uaaaugcauuaucuggAaugu.....................	4	1	Mm2
....uaaaugcauuaucugguaugu.....................	1673	0	Mm2
....uaaauAcauuaucugguaugu.....................	1	1	Mm2
....uaaaCgcauuaucugguaugu.....................	1	1	Mm2
....uGaaugcauuaucugguaugu.....................	1	1	Mm2
....uaaaugcauuaucCgguaugu.....................	2	1	Mm2
....uaGaugcauuaucugguaugu.....................	3	1	Mm2
....uaaaugcauuaucugguaugG.....................	5	1	Mm2
....uaaUugcauuaucugguaugu.....................	1	1	Mm2
....uaaaugcauuUucugguaugu.....................	1	1	Mm2
....uaaaugcauuaucuggCaugu.....................	12	1	Mm2
....uaaaugcauuaucugguaugC.....................	3	1	Mm2
....uaaaugcauuaucuggGaugu.....................	2	1	Mm2
....uaaaugcauuaucugguCugu.....................	6	1	Mm2
....uaaaugcauuaucAgguaugu.....................	1	1	Mm2
....uaaaugcauuaucugguUugu.....................	3	1	Mm2
....uaaGugcauuaucugguaugu.....................	1	1	Mm2
....uaaaugcauuaucugguGugu.....................	8	1	Mm2
....uaaaugcUuuaucugguaugu.....................	1	1	Mm2
....uaaaugcauuCucugguaugu.....................	1	1	Mm2
....uaaaugcauuGucugguaugu.....................	5	1	Mm2
....uaaaugcaCuaucugguaugu.....................	1	1	Mm2
....Aaaaugcauuaucugguaugua....................	1	1	Mm2
....uaaaugcauuaucugguGugua....................	15	1	Mm2
....uaaaugcauuaucugguaugCa....................	3	1	Mm2
....uaUaugcauuaucugguaugua....................	2	1	Mm2
....uaaaugcauuaucugguUugua....................	9	1	Mm2
....uaaaugcauuCucugguaugua....................	8	1	Mm2
....uaaaugcauCaucugguaugua....................	1	1	Mm2
....uaaaugcauuaucCgguaugua....................	1	1	Mm2
....uaaaugcauuaucAgguaugua....................	2	1	Mm2
....uaaaugcauuaucugguaGgua....................	1	1	Mm2
....uaaaugcauuGucugguaugua....................	1	1	Mm2
....Naaaugcauuaucugguaugua....................	4	1	Mm2
....uaaaugcauuaucuUguaugua....................	1	1	Mm2
....uaaaugcauuaucuggGaugua....................	7	1	Mm2
....uaaaugcauuaucugguauguG....................	8	1	Mm2

Star Mature

```
cuagauguuguuuuguugauaccuguggaggguucguaucagaggugcauuugcagugaaguauucuguguaaaugcauuaucugguauguaacucucgcauguugugcagc
......................................................................uaaaugcauuaucugAuaugua....................  1     1  Mm2
......................................................................uCaaugcauuaucugguaugua....................  1     1  Mm2
......................................................................uaaaugcaCuaucugguaugua....................  1     1  Mm2
......................................................................uGaaugcauuaucugguaugua....................  2     1  Mm2
......................................................................uaaaugcCuuaucugguaugua....................  1     1  Mm2
......................................................................uaaaugcauuUucugguaugua....................  3     1  Mm2
......................................................................uUaaugcauuaucugguaugua....................  4     1  Mm2
......................................................................uaaaugcauuaucuggCaugua....................  15    1  Mm2
......................................................................uaaaCgcauuaucugguaugua....................  1     1  Mm2
......................................................................uaaaugcGuuaucugguaugua....................  1     1  Mm2
......................................................................uaaaugcauuaucugguaugua....................  3920  0  Mm2
......................................................................uaaaugcauuaucuggAaugua....................  9     1  Mm2
......................................................................uaaaGgcauuaucugguaugua....................  1     1  Mm2
......................................................................uaaaugcauuaucugguCugua....................  20    1  Mm2
......................................................................uaaaugcauAaucugguaugua....................  1     1  Mm2
......................................................................uaGaugcauuaucugguaugua....................  2     1  Mm2
......................................................................uaaauNcauuaucugguaugua....................  1     1  Mm2
......................................................................uaaaugcauuaucugguauguU....................  39    1  Mm2
......................................................................uaaaugcaAuaucugguaugua....................  1     1  Mm2
......................................................................uaaaugcauuaucuggGauguaa...................  1     1  Mm2
......................................................................uaaauNcauuaucugguauguaa...................  1     1  Mm2
......................................................................uaaaugcauuaucugguUuguaa...................  1     1  Mm2
......................................................................Aaaaugcauuaucugguauguaa...................  1     1  Mm2
......................................................................uaaaugcauuaucugguauguaa...................  342   0  Mm2
......................................................................uaaaugcauuaucugguauguaU...................  34    1  Mm2
......................................................................uaaaugcauuaucugguCuguaa...................  1     1  Mm2
......................................................................uaaaugcauuCucugguauguaa...................  1     1  Mm2
......................................................................uaaaugcauuaucuggAauguaa...................  1     1  Mm2
......................................................................uaaaugcauuGucugguauguaa...................  2     1  Mm2
......................................................................uaaaugcauuaucugguauguaaA..................  3     1  Mm2
......................................................................uaaaugcauuaucugguauguaaU..................  2     1  Mm2
......................................................................uaaaugcauuaucugguauguaaUu.................  1     1  Mm2
.......................................................................aaaugcauuaucugguaugu.....................  3     0  Mm2
.......................................................................aaaugcauuaucugguaugua....................  4     0  Mm2
.......................................................................Uaaugcauuaucugguaugua....................  1     1  Mm2
.......................................................................aaaugcauuaucugguauguaac..................  1     0  Mm2
........................................................................aaugcauuaucugguaGgua....................  1     1  Mm2
.........................................................................augcauuaucugguaugua....................  1     0  Mm2

.....................................................................Cuaaaugcauuaucugguaugua....................  15    1  Fm2
......................................................................uaaaugcauuaucuggua........................  7     0  Fm2
......................................................................uaaaugcauuaucugguau.......................  37    0  Fm2
......................................................................uaaaugcauuaucuggCau.......................  1     1  Fm2
......................................................................uaaaugcGuuaucugguaug......................  1     1  Fm2
......................................................................uaaaGgcauuaucugguaug......................  1     1  Fm2
......................................................................uaaCugcauuaucugguaug......................  1     1  Fm2
......................................................................uaaaugcauuaucuggAaug......................  2     1  Fm2
......................................................................uaaGugcauuaucugguaug......................  1     1  Fm2
......................................................................uaaaugcauuaucuAguaug......................  1     1  Fm2
......................................................................uaaaugcauuauGugguaug......................  1     1  Fm2
......................................................................uCaaugcauuaucugguaug......................  1     1  Fm2
......................................................................uaaaugcauuaucugguCug......................  7     1  Fm2
......................................................................Naaaugcauuaucugguaug......................  2     1  Fm2
......................................................................uaaaugcauuaucuggGaug......................  2     1  Fm2
......................................................................uaaaugcauuaucuggCaug......................  9     1  Fm2
......................................................................uaaaugcauuCucugguaug......................  1     1  Fm2
......................................................................uaaaugcauuaCcugguaug......................  1     1  Fm2
......................................................................uaaaugcauuaucugguUug......................  2     1  Fm2
......................................................................uaaaugcauCaucugguaug......................  1     1  Fm2
......................................................................uaaaugcauuaucugguGug......................  6     1  Fm2
......................................................................uaGaugcauuaucugguaug......................  1     1  Fm2
......................................................................uaaaugcauuaucugguaug......................  1551  0  Fm2
......................................................................uaaaugcauuaGcugguaugu.....................  1     1  Fm2
......................................................................uCaaugcauuaucugguaugu.....................  1     1  Fm2
......................................................................uaaaugcauuaucuUguaugu.....................  2     1  Fm2
......................................................................uaaaugcUuuaucugguaugu.....................  1     1  Fm2
......................................................................uaaaugcauuaucugguUugu.....................  3     1  Fm2
......................................................................uaaauUcauuaucugguaugu.....................  1     1  Fm2
......................................................................uaaaugcauuaucugUuaugu.....................  1     1  Fm2
......................................................................uGaaugcauuaucugguaugu.....................  2     1  Fm2
```

Star **Mature**

cuagauguuguuuuguugauaccuguggaggguucguaucagaggugcauuugcagugaaguauucuguguaaaugcauuaucugguauguaacucucgcauguugugcagc			
....uaaauAcauuaucugguaugu.....................	1	1	Fm2
....uaaaugcauuaucuggCaugu.....................	10	1	Fm2
....uaaaugcauuGucugguaugu.....................	4	1	Fm2
....uaGaugcauuaucugguaugu.....................	1	1	Fm2
....uaaaugcauuaucugguGugu.....................	5	1	Fm2
....uaaUugcauuaucugguaugu.....................	1	1	Fm2
....uaaaugcauuaucugguCugu.....................	7	1	Fm2
....uaaaugAauuaucugguaugu.....................	1	1	Fm2
....uaaaugcauuaucugguaugG.....................	16	1	Fm2
....uaCaugcauuaucugguaugu.....................	1	1	Fm2
....uaaaugcauuCucugguaugu.....................	4	1	Fm2
....uaaaugcaCuaucugguaugu.....................	1	1	Fm2
....uaaaugcauuUucugguaugu.....................	1	1	Fm2
....Naaaugcauuaucugguaugu.....................	2	1	Fm2
....uaaaugcauuaucugguaugu.....................	2991	0	Fm2
....uaaaugcauuauUugguaugu.....................	1	1	Fm2
....uaaaugcauuaucuggGaugu.....................	8	1	Fm2
....uaaaugcauuaucuggAaugu.....................	4	1	Fm2
....uCaaugcauuaucugguaugua....................	3	1	Fm2
....uaaaugcauuGucugguaugua....................	9	1	Fm2
....uaaaugcauuauUugguaugua....................	2	1	Fm2
....uaaaugcauGaucugguaugua....................	1	1	Fm2
....uaaaugcGuuaucugguaugua....................	3	1	Fm2
....uaaaugcauuaAcugguaugua....................	2	1	Fm2
....uaaaugcauuaucuUguaugua....................	4	1	Fm2
....uaaaugcauuaucuggAaugua....................	24	1	Fm2
....uaaaugcauCaucugguaugua....................	1	1	Fm2
....uaaUugcauuaucugguaugua....................	3	1	Fm2
....uaaaugcauuaucugguaAgua....................	1	1	Fm2
....uaaaugcauuaucugguGugua....................	35	1	Fm2
....uaaaugcauuaucCgguaugua....................	1	1	Fm2
....uaaaugcauuCucugguaugua....................	11	1	Fm2
....uaaaugUauuaucugguaugua....................	4	1	Fm2
....uaaaCgcauuaucugguaugua....................	2	1	Fm2
....uaaauUcauuaucugguaugua....................	2	1	Fm2
....uaaauNcauuaucugguaugua....................	1	1	Fm2
....uaaaugcauuaucugguauCua....................	1	1	Fm2
....Aaaaugcauuaucugguaugua....................	2	1	Fm2
....uaaaugcaCuaucugguaugua....................	1	1	Fm2
....uaaaugcauuaucAgguaugua....................	5	1	Fm2
....uaaaugcauuaCcugguaugua....................	1	1	Fm2
....uaaaugcauuaucugguUugua....................	41	1	Fm2
....uaaaugcauuaucugguaugua....................	12108	0	Fm2
....Naaaugcauuaucugguaugua....................	9	1	Fm2
....uaaaugcauuaucugguauguG....................	20	1	Fm2
....uaaaugcauuaucuggCaugua....................	50	1	Fm2
....Caaaugcauuaucugguaugua....................	4	1	Fm2
....uaaaugcauuaucuAguaugua....................	4	1	Fm2
....uaaaugcUuuaucugguaugua....................	4	1	Fm2
....uaaauAcauuaucugguaugua....................	1	1	Fm2
....uaaaugcauuaucugguauguU....................	116	1	Fm2
....uaaaugcauuaucugguauAua....................	2	1	Fm2
....uaaaugcauuaucugguaugCa....................	4	1	Fm2
....uaUaugcauuaucugguaugua....................	2	1	Fm2
....uaaCugcauuaucugguaugua....................	2	1	Fm2
....uaaaAgcauuaucugguaugua....................	1	1	Fm2
....uaaaugcauuaucugguauguC....................	2	1	Fm2
....uaaaugcauuauAgguaugua....................	1	1	Fm2
....uaaaugcauuaucuCguaugua....................	2	1	Fm2
....uaaaugcauuaucugguaCgua....................	1	1	Fm2
....uaaaugcauuaGcugguaugua....................	1	1	Fm2
....uaaaugGauuaucugguaugua....................	2	1	Fm2
....uaaaugcaAuaucugguaugua....................	3	1	Fm2
....uaGaugcauuaucugguaugua....................	5	1	Fm2
....uaaaugcauuUucugguaugua....................	8	1	Fm2
....uaaaugcauuaucugguauUua....................	2	1	Fm2
....uaaaugcauuaucuggGaugua....................	31	1	Fm2
....uaaaugcauuaucugUuaugua....................	3	1	Fm2
....uaaaugcauuaucugguCugua....................	44	1	Fm2
....uaaaugcauuaucGgguaugua....................	1	1	Fm2

Star Mature

cuagauguuguuuuguugauaccuguggaggguucguaucagaggugcauuugcagugaaguauucuguguaaaugcauuaucugguauguaacucucgcauguugugcagc			
....uaaaugcauuaucuggAauguaa...................	2	1	Fm2
....uaaaugcauuaucugguUuguaa...................	3	1	Fm2
....uaaauNcauuaucugguauguaa...................	1	1	Fm2
....uaaaugcauuaucugguauguaa...................	773	0	Fm2
....uaaaugcauuUucugguauguaa...................	3	1	Fm2
....uaaaugcauuaucugguCuguaa...................	1	1	Fm2
....uaaaugcauuaucugguauguaG...................	9	1	Fm2
....uaaaugcauuGucugguauguaa...................	1	1	Fm2
....uaaaugcauuaucugguGuguaa...................	5	1	Fm2
....uaaaugcauuaucugguauguaU...................	101	1	Fm2
....uaaaugcauuaucugguauguaaU..................	17	1	Fm2
....aaaugcauuaucugguaug......................	8	0	Fm2
....aaaugcauuaGcugguaugu.....................	1	1	Fm2
....aaaugcauuaucugguaugu.....................	2	0	Fm2
....aaaugcauuaucugguaugua....................	5	0	Fm2
....Uaaugcauuaucugguaugua....................	1	1	Fm2
....aaugcauuaucugguaugua....................	4	0	Fm2
....aaugcauuaucugguauguaa...................	9	0	Fm2
.................................ucguaucagaggugcauuug...	3	0	Fm1
.................................ucguaucagaggugcauuugc..	9	0	Fm1
..................................cguaucagaggugcauuug...	4	0	Fm1
..................................cguaucagaggugcauuugc..	8	0	Fm1
..................................cguaucagaggugcauuugca...	11	0	Fm1
....Cuaaaugcauuaucugguaugua....................	3	1	Fm1
....uaaaugcauuaucuggua........................	3	0	Fm1
....uaaaugcauuaucugguU........................	5	1	Fm1
....uaaaugcauuaucugguaA.......................	1	1	Fm1
....uaaaugcauuaucugguGu.......................	1	1	Fm1
....uaaaugcauuaucugguau.......................	38	0	Fm1
....uaaaugcauuaucuggGau.......................	1	1	Fm1
....uaaaugcauuaucugguUu.......................	3	1	Fm1
....uaaaugcGuuaucugguaug......................	1	1	Fm1
....uaaaugcauuaucugguGug......................	4	1	Fm1
....uaaaugcauuGucugguaug......................	3	1	Fm1
....uaaaugcauuaucuggGaug......................	2	1	Fm1
....uaaaugcauuaucugguaGg......................	3	1	Fm1
....uaGaugcauuaucugguaug......................	1	1	Fm1
....uaaaugcauuauUugguaug......................	2	1	Fm1
....uaaauUcauuaucugguaug......................	1	1	Fm1
....uaaaugcauuaucugguauA......................	1	1	Fm1
....uaaaugcaAuaucugguaug......................	1	1	Fm1
....uaaaugcauuaucuggAaug......................	2	1	Fm1
....uaaaugcauuaucugguauU......................	4	1	Fm1
....uaaaugcauuaucugguCug......................	3	1	Fm1
....uaaaugcauuaucuggCaug......................	2	1	Fm1
....uaaaugcauuaucugguaug......................	1198	0	Fm1
....uaaaugcauuaucugAuaug......................	1	1	Fm1
....uaaaugcauuaucugguUug......................	2	1	Fm1
....uaaaugcauuaucuggCaugu.....................	9	1	Fm1
....uaaaugcaGuaucugguaugu.....................	1	1	Fm1
....uaaaugcauuaCcugguaugu.....................	1	1	Fm1
....Caaaugcauuaucugguaugu.....................	1	1	Fm1
....uaaaugcauuaucugguaugG.....................	15	1	Fm1
....uaaaugcauuaucuggGaugu.....................	5	1	Fm1
....Naaaugcauuaucugguaugu.....................	3	1	Fm1
....uaaaugAauuaucugguaugu.....................	1	1	Fm1
....uaGaugcauuaucugguaugu.....................	1	1	Fm1
....uaaaugcauuauUugguaugu.....................	2	1	Fm1
....uaaaugcauuaucugguauUu.....................	2	1	Fm1
....uaaaugcauuaucugguCugu.....................	15	1	Fm1
....uaaaugcauuCucugguaugu.....................	4	1	Fm1
....uaaaugcauuaucGgguaugu.....................	1	1	Fm1
....uaaaugcauuaucugguGugu.....................	7	1	Fm1
....uaaaugcauuGucugguaugu.....................	6	1	Fm1
....uaaaugNauuaucugguaugu.....................	1	1	Fm1
....uaaaugcauuaucugguaCgu.....................	1	1	Fm1
....uaCaugcauuaucugguaugu.....................	1	1	Fm1
....uUaaugcauuaucugguaugu.....................	2	1	Fm1
....uaaCugcauuaucugguaugu.....................	1	1	Fm1

Star Mature

cuagauguuguuuuguugauaccuguggaggguucguaucagaggugcauuugcagugaaguauucuguguaaaugcauuaucugguauguaacucucgcauguugugcagc			
....uaaaugcUuuaucugguaugu.....................	4	1	Fm1
....uaaaugcauuaucuggAaugu.....................	2	1	Fm1
....uaaaugcauuaucugguaugu.....................	3328	0	Fm1
....uaaaugcauuaucugguauAu.....................	2	1	Fm1
....uaaUugcauuaucugguaugu.....................	2	1	Fm1
....uaaaugcauuaucugguUugu.....................	7	1	Fm1
....uaaauUcauuaucugguaugu.....................	1	1	Fm1
....uaaaugcauGaucugguaugua....................	3	1	Fm1
....uaaaugcauuaucugguauguC....................	4	1	Fm1
....uaaaugcauuaucugCuaugua....................	1	1	Fm1
....uaaaugcauuaucugguaGgua....................	1	1	Fm1
....uaaauUcauuaucugguaugua....................	3	1	Fm1
....uaaaugcGuuaucugguaugua....................	7	1	Fm1
....uaaauAcauuaucugguaugua....................	1	1	Fm1
....Aaaaugcauuaucugguaugua....................	5	1	Fm1
....uaaaCgcauuaucugguaugua....................	1	1	Fm1
....uaaaugcauuUucugguaugua....................	9	1	Fm1
....uGaaugcauuaucugguaugua....................	1	1	Fm1
....uaaaGgcauuaucugguaugua....................	1	1	Fm1
....uUaaugcauuaucugguaugua....................	7	1	Fm1
....Caaaugcauuaucugguaugua....................	11	1	Fm1
....uaaaugcauuaucugguauguU....................	101	1	Fm1
....uaaaugcauuaucuggGaugua....................	36	1	Fm1
....uaaaugAauuaucugguaugua....................	1	1	Fm1
....uaaaugcauuaucugguauAua....................	4	1	Fm1
....Naaaugcauuaucugguaugua....................	7	1	Fm1
....uaaaugcauuaucugguCugua....................	45	1	Fm1
....uaaaugcauuaucugguauguG....................	15	1	Fm1
....uaaaugcauuCucugguaugua....................	15	1	Fm1
....uaaCugcauuaucugguaugua....................	3	1	Fm1
....uaaaugcauuGucugguaugua....................	18	1	Fm1
....uaaaugUauuaucugguaugua....................	2	1	Fm1
....uaaaugcaCuaucugguaugua....................	2	1	Fm1
....uaaaugcauuaucugguaAgua....................	1	1	Fm1
....uaaaugcauuaucuggAaugua....................	29	1	Fm1
....uaCaugcauuaucugguaugua....................	3	1	Fm1
....uaaaugcauuaucuCguaugua....................	1	1	Fm1
....uaaaugcauuaucuggCaugua....................	60	1	Fm1
....uaaaugcauuaucAgguaugua....................	5	1	Fm1
....uaaaugcUuuaucugguaugua....................	4	1	Fm1
....uaaaugcauuaucugUuaugua....................	3	1	Fm1
....uaaaugcauuaucCgguaugua....................	3	1	Fm1
....uaaaugcauuaucugguaugAa....................	3	1	Fm1
....uaaaugcauuaucugguaugCa....................	8	1	Fm1
....uaaaugcauuaucugguUugua....................	42	1	Fm1
....uaUaugcauuaucugguaugua....................	1	1	Fm1
....uaGaugcauuaucugguaugua....................	4	1	Fm1
....uaaauNcauuaucugguaugua....................	1	1	Fm1
....uaaaugcauuaucugguGugua....................	33	1	Fm1
....uaaaugGauuaucugguaugua....................	1	1	Fm1
....uaaaugcauuaucuUguaugua....................	1	1	Fm1
....uaaUugcauuaucugguaugua....................	2	1	Fm1
....uaaaugcauuaCcugguaugua....................	1	1	Fm1
....uaaaugcaGuaucugguaugua....................	1	1	Fm1
....uaaaAgcauuaucugguaugua....................	1	1	Fm1
....uaaaugcauuaucugguaugua....................	12607	0	Fm1
....uGaaugcauuaucugguauguaa...................	1	1	Fm1
....uaaaugcauuaucugguauguaU...................	137	1	Fm1
....uaaaugcauuGucugguauguaa...................	1	1	Fm1
....uaaaugcauuaucugguUuguaa...................	3	1	Fm1
....uaaaugcauuaucugguGuguaa...................	1	1	Fm1
....uaaaugcauuaucugguCuguaa...................	2	1	Fm1
....uaaaugcauuaucugguauguaa...................	693	0	Fm1
....uaaaugcGuuaucugguauguaa...................	2	1	Fm1
....uaaaugcauuaucuggGauguaa...................	2	1	Fm1
....uaaaugcauuaucugguauUuaa...................	1	1	Fm1
....uaaaugcauuaucugguauguaaU..................	9	1	Fm1
....uaaaugcauuaucugguauguaac..................	2	0	Fm1
....uaaaugcauuaucugguauguaaG..................	1	1	Fm1
....uaaaugcauuaucugguauguaaA..................	2	1	Fm1

Star **Mature**

```
cuagauguuguuuuguugauaccuguggagguucguaucagaggugcauuugcagugaaguauucuguguaaaugcauuaucugguauguaacucucgcauguugugcagc
......................................................................aaaugcauuaucugguaug......................   1      0      Fm1
......................................................................aaaugcauuaucugguauUu....................   1      1      Fm1
......................................................................aaaugcauuaucugguaugu.....................   6      0      Fm1
......................................................................Uaaugcauuaucugguaugu.....................   1      1      Fm1
......................................................................aaaugcauuaucugguaugAa....................   1      1      Fm1
......................................................................aaaugcauuaucugguaugua....................   16     0      Fm1
......................................................................Uaaugcauuaucugguaugua....................   2      1      Fm1
.......................................................................aaugcauuaucugguaugua....................   2      0      Fm1
.......................................................................aaugcauuaucugguauguaa...................   1      0      Fm1

................................ucguaucagaggugcauuugc..........................................................   4      0      Mm3
.................................cguaucagaggugcauuugc..........................................................   1      0      Mm3
.................................cguaucagaggugcauuGgc..........................................................   1      1      Mm3
.................................cguaucagaggugcauuCgca.........................................................   1      1      Mm3
.................................cguaucagaggugcauuugca.........................................................   6      0      Mm3
.................................cguaucagaggugcauuugcagugaagu..................................................   1      0      Mm3
....................................................................Cuaaaugcauuaucugguaugu.....................   2      1      Mm3
.....................................................................uaaaugcauuaucugguC........................   1      1      Mm3
.....................................................................uaaaugcauuaucuggua........................   6      0      Mm3
.....................................................................uaaaugcauuaucugguau.......................   38     0      Mm3
.....................................................................uaaaugcauuaucugguCug......................   2      1      Mm3
.....................................................................uaaaugcauuaucuggGaug......................   1      1      Mm3
.....................................................................uaaaugcauuaucugguauU......................   2      1      Mm3
.....................................................................uaaaugcauuaucugAuaug......................   1      1      Mm3
.....................................................................uaaaugcauuaucugguaug......................   415    0      Mm3
.....................................................................uaaGugcauuaucugguaug......................   1      1      Mm3
.....................................................................uaaaugcauuaucugguGug......................   2      1      Mm3
.....................................................................uaaaugcauuaucuggCaug......................   5      1      Mm3
.....................................................................uaaaugcCuuaucugguaugu.....................   1      1      Mm3
.....................................................................uaaaugcauuaucugguUugu.....................   7      1      Mm3
.....................................................................uUaaugcauuaucugguaugu.....................   1      1      Mm3
.....................................................................uaaaugcauuaucugguauUu.....................   1      1      Mm3
.....................................................................uaaaugcauuGucugguaugu.....................   1      1      Mm3
.....................................................................uaaauUcauuaucugguaugu.....................   1      1      Mm3
.....................................................................uaaaugcauuCucugguaugu.....................   2      1      Mm3
.....................................................................uaaaugcauuaucugguGugu.....................   7      1      Mm3
.....................................................................uaUaugcauuaucugguaugu.....................   1      1      Mm3
.....................................................................uaaaugcauuaucAgguaugu.....................   1      1      Mm3
.....................................................................uaaaugcauuaucuUguaugu.....................   1      1      Mm3
.....................................................................uaaaugcauuaucuggAaugu.....................   8      1      Mm3
.....................................................................uaaaugcauuaucuggGaugu.....................   3      1      Mm3
.....................................................................uaaaugcauuaucugguaugu.....................   2669   0      Mm3
.....................................................................uaaaugcauuaucuggCaugu.....................   5      1      Mm3
.....................................................................uaaaugcauuaucugguaugG.....................   13     1      Mm3
.....................................................................uaaGugcauuaucugguaugu.....................   1      1      Mm3
.....................................................................uaaCugcauuaucugguaugu.....................   1      1      Mm3
.....................................................................uaaaugcauuUucugguaugu.....................   2      1      Mm3
.....................................................................Naaaugcauuaucugguaugu.....................   1      1      Mm3
.....................................................................uaaaugNauuaucugguaugu.....................   1      1      Mm3
.....................................................................uaaaugcaCuaucugguaugu.....................   1      1      Mm3
.....................................................................uaaaugcauuaAcugguaugu.....................   1      1      Mm3
.....................................................................uaaaugcauuaucugguaugC.....................   2      1      Mm3
.....................................................................uaaaugUauuaucugguaugu.....................   1      1      Mm3
.....................................................................uaaaugcauuaucCgguaugu.....................   1      1      Mm3
.....................................................................uaaaugcauAaucugguaugu.....................   1      1      Mm3
.....................................................................uaaaugcauuaucugguCugu.....................   10     1      Mm3
.....................................................................uaaaugcauuaucugUuaugu.....................   1      1      Mm3
.....................................................................uaaaugcauuaucugguaugua....................   8395   0      Mm3
.....................................................................uaaaugcauuaucugguUugua....................   21     1      Mm3
.....................................................................uaaaugcauuUucugguaugua....................   3      1      Mm3
.....................................................................uaaaugcauuauAugguaugua....................   1      1      Mm3
.....................................................................uaaaugcauuaucuggGaugua....................   14     1      Mm3
.....................................................................uaaaugUauuaucugguaugua....................   1      1      Mm3
.....................................................................uaaaugcauuaucugguGugua....................   25     1      Mm3
.....................................................................uaaaugcauuaucuggAaugua....................   12     1      Mm3
.....................................................................uaaaugcauuaucugguauguU....................   104    1      Mm3
.....................................................................uaaUugcauuaucugguaugua....................   4      1      Mm3
.....................................................................uaaaugcauuaucugguauAua....................   1      1      Mm3
.....................................................................uaaaugcauuaucCgguaugua....................   2      1      Mm3
.....................................................................uaaaugcauGaucugguaugua....................   1      1      Mm3
```

Star Mature

```
cuagauguuguuuuguugauaccuguggaggguucguaucagaggugcauuugcagugaaguauucuguguaaaugcauuaucugguauguaacucucgcauguugugcagc
......................................................................uaaaugcauuaucAgguaugua....................   1    1  Mm3
......................................................................uGaaugcauuaucugguaugua....................   1    1  Mm3
......................................................................uaaaugcauAaucugguaugua....................   3    1  Mm3
......................................................................uaaaAgcauuaucugguaugua....................   1    1  Mm3
......................................................................uaaaCgcauuaucugguaugua....................   1    1  Mm3
......................................................................uaaaugcaAuaucugguaugua....................   1    1  Mm3
......................................................................Caaaugcauuaucugguaugua....................   3    1  Mm3
......................................................................uaaaugcauuaucuggCaugua....................   24   1  Mm3
......................................................................uaaaugcauuaucugCuaugua....................   1    1  Mm3
......................................................................uaCaugcauuaucugguaugua....................   2    1  Mm3
......................................................................uaaGugcauuaucugguaugua....................   3    1  Mm3
......................................................................Naaaugcauuaucugguaugua....................   6    1  Mm3
......................................................................uaaaugcauuaucugguauUua....................   2    1  Mm3
......................................................................uaaCugcauuaucugguaugua....................   1    1  Mm3
......................................................................uaaaugcauuaucugAuaugua....................   1    1  Mm3
......................................................................uaaaugNauuaucugguaugua....................   1    1  Mm3
......................................................................uaaauAcauuaucugguaugua....................   1    1  Mm3
......................................................................uaaaugAauuaucugguaugua....................   1    1  Mm3
......................................................................uaaauUcauuaucugguaugua....................   2    1  Mm3
......................................................................uaaaugcauuaucugguaugAa....................   2    1  Mm3
......................................................................uaaauNcauuaucugguaugua....................   1    1  Mm3
......................................................................uUaaugcauuaucugguaugua....................   3    1  Mm3
......................................................................uaaaugcauuGucugguaugua....................   19   1  Mm3
......................................................................uaaaugcauuaucugguCugua....................   36   1  Mm3
......................................................................uaaaugcauuaucugguaugCa....................   14   1  Mm3
......................................................................uaaaugcauuaucugguaAgua....................   1    1  Mm3
......................................................................uaaaugcauuaucugguauguG....................   6    1  Mm3
......................................................................uaaaugcauuCucugguaugua....................   3    1  Mm3
......................................................................uaaaugcauuaucGgguaugua....................   1    1  Mm3
......................................................................uaUaugcauuaucugguaugua....................   1    1  Mm3
......................................................................uaaaugcUuuaucugguaugua....................   3    1  Mm3
......................................................................uaaaugcauuaucugUuaugua....................   2    1  Mm3
......................................................................uaaaugcauuaucugguCuguaa...................   2    1  Mm3
......................................................................uaaaugcauuaucugguGuguaa...................   1    1  Mm3
......................................................................uaaaugcauuauUugguauguaa...................   1    1  Mm3
......................................................................uaaaugcauuaucuggAauguaa...................   1    1  Mm3
......................................................................uaaaugcauuaucuggGauguaa...................   2    1  Mm3
......................................................................uaaaugcauuaucugguauguaa...................   531  0  Mm3
......................................................................uaaaugcauuaucugguUuguaa...................   2    1  Mm3
......................................................................Caaaugcauuaucugguauguaa...................   1    1  Mm3
......................................................................uaaaugNauuaucugguauguaa...................   1    1  Mm3
......................................................................uaaaugcauuGucugguauguaa...................   1    1  Mm3
......................................................................uaaaugcauuUucugguauguaa...................   1    1  Mm3
......................................................................uaaaugcauuaucugguauguaU...................   60   1  Mm3
......................................................................uaaaugcauuaucugguauguaaA..................   4    1  Mm3
......................................................................uaaaugcauuaucugguauguaac..................   3    0  Mm3
......................................................................uaaaugcauuaucugguauguaaU..................   9    1  Mm3
......................................................................uaaaugcauuaucugguauguaaUu.................   1    1  Mm3
.......................................................................aaaugcauuaAcugguaugu.....................   1    1  Mm3
.......................................................................aaaugcauuaucugguaugu.....................   2    0  Mm3
.......................................................................aaaugcauuaucugguaugua....................   18   0  Mm3
.......................................................................aaaugcauuaucugguauguaa...................   1    0  Mm3
.........................................................................augcauuaucugguaugua....................   1    0  Mm3

.................................ucguaucagaggugcauuug...........................................................   2    0  Fm3
.................................ucguaucagaggugcauuugc..........................................................   6    0  Fm3
.................................ucguaucagaggugcauuugca.........................................................   3    0  Fm3
..................................cguaucagaggugcauuug...........................................................   3    0  Fm3
..................................Uguaucagaggugcauuugc..........................................................   1    1  Fm3
..................................cguaucagaggugcauuugc..........................................................   30   0  Fm3
..................................cguaucagaggugcauuugca.........................................................   15   0  Fm3
..................................cguaucagaggugcauGugca.........................................................   1    1  Fm3
..................................cguaucagaggugcauuCgca.........................................................   1    1  Fm3
...................................guaucagaggugcauuugc..........................................................   2    0  Fm3
.....................................................................Cuaaaugcauuaucugguaug......................   2    1  Fm3
.....................................................................Cuaaaugcauuaucugguaugu.....................   5    1  Fm3
.....................................................................Cuaaaugcauuaucugguaugua....................   11   1  Fm3
......................................................................uaaaugcauuaucugguU........................   6    1  Fm3
......................................................................uaaaugcauuaucugguG........................   1    1  Fm3
......................................................................uaaaugcauuaucuggua........................   22   0  Fm3
```

```
                                  Star                                Mature
cuagauguuguuuuguugauaccuguggaggguucguaucagaggugcauuugcagugaaguauucuguguaaaugcauuaucugguauguaacucucgcauguugugcagc
......................................................................uaaCugcauuaucugguau........................  1      1  Fm3
......................................................................uaaaugcGuuaucugguau........................  1      1  Fm3
......................................................................uaaaugcauuGucugguau........................  1      1  Fm3
......................................................................uaaaugcauuaucugguau........................  212    0  Fm3
......................................................................uaaaugcauuaucuggCau........................  1      1  Fm3
......................................................................uaaaugcauuaucugguaA........................  7      1  Fm3
......................................................................uaaaugcauuCucugguau........................  2      1  Fm3
......................................................................uaaaugcauuaucugUuau........................  1      1  Fm3
......................................................................uaaaugcauuaucuggAau........................  1      1  Fm3
......................................................................uaaaugcauuaucuggCaug.......................  45     1  Fm3
......................................................................uaaaugcauuaAcugguaug.......................  2      1  Fm3
......................................................................uaaaugAauuaucugguaug.......................  1      1  Fm3
......................................................................uaaaugcauuaucugguGug.......................  42     1  Fm3
......................................................................uaaaugNauuaucugguaug.......................  1      1  Fm3
......................................................................uaaaugcauuauUugguaug.......................  16     1  Fm3
......................................................................uaaaugcauuaucugguCug.......................  57     1  Fm3
......................................................................uaaaugcauGaucugguaug.......................  1      1  Fm3
......................................................................uaaaugcauuCucugguaug.......................  13     1  Fm3
......................................................................uaaaugcauuaucugguauA.......................  6      1  Fm3
......................................................................uaaaugcauuaucuggAaug.......................  29     1  Fm3
......................................................................uaaaugcauuaucugguaAg.......................  3      1  Fm3
......................................................................uaCaugcauuaucugguaug.......................  1      1  Fm3
......................................................................uaaaugcauuaucugguaug.......................  11685  0  Fm3
......................................................................uaaaAgcauuaucugguaug.......................  4      1  Fm3
......................................................................uaaaugcauuGucugguaug.......................  17     1  Fm3
......................................................................uUaaugcauuaucugguaug.......................  3      1  Fm3
......................................................................uaUaugcauuaucugguaug.......................  1      1  Fm3
......................................................................uaaaugcauuaucugguaCg.......................  1      1  Fm3
......................................................................uaaaugcauuaucuCguaug.......................  2      1  Fm3
......................................................................uaGaugcauuaucugguaug.......................  6      1  Fm3
......................................................................uaaUugcauuaucugguaug.......................  4      1  Fm3
......................................................................uaaaugcauCaucugguaug.......................  1      1  Fm3
......................................................................uaaaugcauuUucugguaug.......................  5      1  Fm3
......................................................................uaaaugcauuaucuUguaug.......................  7      1  Fm3
......................................................................uaaaugcauuaucugAuaug.......................  1      1  Fm3
......................................................................uaaaugcauuaucugguUug.......................  35     1  Fm3
......................................................................uaaGugcauuaucugguaug.......................  3      1  Fm3
......................................................................uaaaugcUuuaucugguaug.......................  3      1  Fm3
......................................................................uaaaugcauuaucGgguaug.......................  1      1  Fm3
......................................................................uaaCugcauuaucugguaug.......................  1      1  Fm3
......................................................................uaaauUcauuaucugguaug.......................  3      1  Fm3
......................................................................uaaaugcauuaucugguauU.......................  9      1  Fm3
......................................................................uaaaCgcauuaucugguaug.......................  5      1  Fm3
......................................................................uaaaugcGuuaucugguaug.......................  2      1  Fm3
......................................................................uaaauNcauuaucugguaug.......................  3      1  Fm3
......................................................................uaaaugUauuaucugguaug.......................  4      1  Fm3
......................................................................uaaaugcauuaucuggGaug.......................  24     1  Fm3
......................................................................uaaaugcauuaucuAguaug.......................  2      1  Fm3
......................................................................Naaaugcauuaucugguaug.......................  5      1  Fm3
......................................................................uaaaugcauuaucAgguaug.......................  3      1  Fm3
......................................................................uaaauUcauuaucugguaugu......................  2      1  Fm3
......................................................................Gaaaugcauuaucugguaugu......................  2      1  Fm3
......................................................................uaaGugcauuaucugguaugu......................  6      1  Fm3
......................................................................uaaaugcauuaucugCuaugu......................  1      1  Fm3
......................................................................uaaaugcauuaucugAuaugu......................  3      1  Fm3
......................................................................uaaaugcauuaucugguaugC......................  1      1  Fm3
......................................................................uaaaugcauuaucugguauUu......................  1      1  Fm3
......................................................................uaaaugcauuaucugguCugu......................  117    1  Fm3
......................................................................uaaaugcauuaucugguaCgu......................  3      1  Fm3
......................................................................uaaaugcauCaucugguaugu......................  5      1  Fm3
......................................................................uaaaugcauuaucuggGaugu......................  74     1  Fm3
......................................................................uaaaugcauuUucugguaugu......................  19     1  Fm3
......................................................................uaaaugcauuaucuCguaugu......................  3      1  Fm3
......................................................................uaaaugcGuuaucugguaugu......................  9      1  Fm3
......................................................................uaaaugcauuaucugguaAgu......................  2      1  Fm3
......................................................................uaCaugcauuaucugguaugu......................  1      1  Fm3
......................................................................uaaaugcauuCucugguaugu......................  21     1  Fm3
......................................................................uaaaugcauuaucuAguaugu......................  9      1  Fm3
......................................................................uaaauNcauuaucugguaugu......................  2      1  Fm3
......................................................................uaaaugcaCuaucugguaugu......................  2      1  Fm3
```

```
                                  Star                                    Mature
cuagauguuguuuuguugauaccuguggaggguucguaucagaggugcauuugcagugaaguauucugugugaaaugcauuaucugguauguaacucucgcauguugugcagc
......................................................................uaaaCgcauuaucugguaugu.....................    8      1    Fm3
......................................................................uaaaugcauuaucCgguaugu.....................    5      1    Fm3
......................................................................uaUaugcauuaucugguaugu.....................    3      1    Fm3
......................................................................uaaCugcauuaucugguaugu.....................    3      1    Fm3
......................................................................Caaaugcauuaucugguaugu.....................    3      1    Fm3
......................................................................uaaaugcauuauAugguaugu.....................    1      1    Fm3
......................................................................uaaaugcauuauGugguaugu.....................    1      1    Fm3
......................................................................uaaaAgcauuaucugguaugu.....................    2      1    Fm3
......................................................................uaaaugcauuGucugguaugu.....................    31     1    Fm3
......................................................................uaaaugcauuaucugguaugA.....................    9      1    Fm3
......................................................................uaaaugcauuaucuggAaugu.....................    67     1    Fm3
......................................................................uaNaugcauuaucugguaugu.....................    1      1    Fm3
......................................................................uCaaugcauuaucugguaugu.....................    3      1    Fm3
......................................................................uaaaugcauuaucugguGugu.....................    63     1    Fm3
......................................................................uaaaugcauuaAcugguaugu.....................    3      1    Fm3
......................................................................uaaaugNauuaucugguaugu.....................    1      1    Fm3
......................................................................uaaaugcauGaucugguaugu.....................    3      1    Fm3
......................................................................uaaaugcauuaucugUuaugu.....................    4      1    Fm3
......................................................................uaaaugcauuaucGgguaugu.....................    2      1    Fm3
......................................................................uaaaugcauuaucugguaugu.....................    26247  0    Fm3
......................................................................uGaaugcauuaucugguaugu.....................    5      1    Fm3
......................................................................uaaaugcauuaucugguaugG.....................    1      1    Fm3
......................................................................uaaaugcauuaCcugguaugu.....................    4      1    Fm3
......................................................................uaaaugcUuuaucugguaugu.....................    4      1    Fm3
......................................................................uaaaGgcauuaucugguaugu.....................    1      1    Fm3
......................................................................Naaaugcauuaucugguaugu.....................    11     1    Fm3
......................................................................uUaaugcauuaucugguaugu.....................    6      1    Fm3
......................................................................uaaaugUauuaucugguaugu.....................    6      1    Fm3
......................................................................uaaaugcauuaucugguauAu.....................    7      1    Fm3
......................................................................uaaaugAauuaucugguaugu.....................    4      1    Fm3
......................................................................uaaaugcauuaucugguauCu.....................    1      1    Fm3
......................................................................uaaaugcauuauUugguaugu.....................    8      1    Fm3
......................................................................uaaaugcauuaucuUguaugu.....................    11     1    Fm3
......................................................................uaaaugcauuaucAgguaugu.....................    7      1    Fm3
......................................................................Aaaaugcauuaucugguaugu.....................    2      1    Fm3
......................................................................uaGaugcauuaucugguaugu.....................    9      1    Fm3
......................................................................uaaaugcauuaucuggCaugu.....................    107    1    Fm3
......................................................................uaaaugcauuaucugguUugu.....................    77     1    Fm3
......................................................................uaaauAcauuaucugguaugu.....................    1      1    Fm3
......................................................................uaaaugcaAuaucugguaugu.....................    4      1    Fm3
......................................................................uaaaugcauuaucugguauguC....................    2      1    Fm3
......................................................................uaaaugNauuaucugguaugua....................    2      1    Fm3
......................................................................uaaaugcauuaucugUuaugua....................    12     1    Fm3
......................................................................uNaaugcauuaucugguaugua....................    1      1    Fm3
......................................................................uaaaugcauuaucuCguaugua....................    6      1    Fm3
......................................................................uaaaugcauuaucugCuaugua....................    8      1    Fm3
......................................................................uaaaugcauuauAugguaugua....................    8      1    Fm3
......................................................................uaaauUcauuaucugguaugua....................    15     1    Fm3
......................................................................uGaaugcauuaucugguaugua....................    28     1    Fm3
......................................................................uaaaugcauuaucugguauguG....................    33     1    Fm3
......................................................................uaaaugcauuaucugAuaugua....................    12     1    Fm3
......................................................................uaaaAgcauuaucugguaugua....................    5      1    Fm3
......................................................................uaaaugcauuaucugguUugua....................    241    1    Fm3
......................................................................uaaaugcauuaucugguaugAa....................    11     1    Fm3
......................................................................uaaaugcauAaucugguaugua....................    1      1    Fm3
......................................................................uaaaugcauuCucugguaugua....................    69     1    Fm3
......................................................................Caaaugcauuaucugguaugua....................    20     1    Fm3
......................................................................uaaaugcauuaucugguaGgua....................    3      1    Fm3
......................................................................uaGaugcauuaucugguaugua....................    33     1    Fm3
......................................................................uaaaugcauuauUugguaugua....................    22     1    Fm3
......................................................................uaaaugAauuaucugguaugua....................    3      1    Fm3
......................................................................uaaaugcauuaucCgguaugua....................    28     1    Fm3
......................................................................uaaaugcauuaucugguCugua....................    339    1    Fm3
......................................................................uUaaugcauuaucugguaugua....................    22     1    Fm3
......................................................................uaaaugcauuaucuUguaugua....................    28     1    Fm3
......................................................................uaaaugcGuuaucugguaugua....................    18     1    Fm3
......................................................................uaaCugcauuaucugguaugua....................    9      1    Fm3
......................................................................Aaaaugcauuaucugguaugua....................    1      1    Fm3
......................................................................uaaaugcauuaCcugguaugua....................    6      1    Fm3
......................................................................uaUaugcauuaucugguaugua....................    15     1    Fm3
```

```
                                                                   Star                    Mature
cuagauguuguuuuguugauaccuguggaggguucguaucagaggugcauuugcagugaaguauucuguguaaaugcauuaucugguauguaacucucgcauguugugcagc
......................................................................uaaauCcauuaucugguaugua....................    3        1    Fm3
......................................................................uaaaugcauuUucugguaugua....................    57       1    Fm3
......................................................................uaaaugcauuaAcugguaugua....................    3        1    Fm3
......................................................................uaaGugcauuaucugguaugua....................    15       1    Fm3
......................................................................uCaaugcauuaucugguaugua....................    12       1    Fm3
......................................................................Gaaaugcauuaucugguaugua....................    1        1    Fm3
......................................................................uaaaugcUuuaucugguaugua....................    35       1    Fm3
......................................................................uaaaugcauGaucugguaugua....................    11       1    Fm3
......................................................................uaaUugcauuaucugguaugua....................    10       1    Fm3
......................................................................uaCaugcauuaucugguaugua....................    12       1    Fm3
......................................................................uaaaugcauuaucugguauguU....................    865      1    Fm3
......................................................................uaaaugcauuaucugguaAgua....................    4        1    Fm3
......................................................................uaaaugcauuaucugguauCua....................    2        1    Fm3
......................................................................uaaaugUauuaucugguaugua....................    17       1    Fm3
......................................................................uaaaGgcauuaucugguaugua....................    2        1    Fm3
......................................................................uaaaugcauCaucugguaugua....................    4        1    Fm3
......................................................................uaaaugcauuaucugguaCgua....................    5        1    Fm3
......................................................................uaaaugcaGuaucugguaugua....................    3        1    Fm3
......................................................................uaaaugcaAuaucugguaugua....................    10       1    Fm3
......................................................................uaaaugcauuaucugguaugua....................    87613    0    Fm3
......................................................................uaaaugcauuaucuggAaugua....................    180      1    Fm3
......................................................................uaaaugcCuuaucugguaugua....................    1        1    Fm3
......................................................................uaaauNcauuaucugguaugua....................    15       1    Fm3
......................................................................uaaaugcauuaucuggGaugua....................    279      1    Fm3
......................................................................uaNaugcauuaucugguaugua....................    1        1    Fm3
......................................................................uaaaugcauuaucAgguaugua....................    19       1    Fm3
......................................................................uaaaugcauuaucGgguaugua....................    8        1    Fm3
......................................................................uaaaugcauuaucugguGugua....................    321      1    Fm3
......................................................................uaaaugcauuaucuggCaugua....................    267      1    Fm3
......................................................................uaaaugcauuaucugguauAua....................    11       1    Fm3
......................................................................uaaaugcauuaucuAguaugua....................    20       1    Fm3
......................................................................uaaaugcauuGucugguaugua....................    129      1    Fm3
......................................................................uaaaugcaCuaucugguaugua....................    12       1    Fm3
......................................................................Naaaugcauuaucugguaugua....................    54       1    Fm3
......................................................................uaaaCgcauuaucugguaugua....................    31       1    Fm3
......................................................................uaaauAcauuaucugguaugua....................    7        1    Fm3
......................................................................uaaaugcauuaucugguaugCa....................    12       1    Fm3
......................................................................uaaaugcauuaucugguauUua....................    14       1    Fm3
......................................................................uaaaugGauuaucugguaugua....................    2        1    Fm3
......................................................................uaaaugcauuaucugguauguaG...................    1        1    Fm3
......................................................................uaaaugAauuaucugguauguaa...................    1        1    Fm3
......................................................................uaaaugcauuaucuggGauguaa...................    12       1    Fm3
......................................................................uUaaugcauuaucugguauguaa...................    1        1    Fm3
......................................................................uaaaugcUuuaucugguauguaa...................    1        1    Fm3
......................................................................uaaaugcauuaucuUguauguaa...................    1        1    Fm3
......................................................................uaaaugcauuaucuAguauguaa...................    1        1    Fm3
......................................................................uaaaugcauuaucCgguauguaa...................    1        1    Fm3
......................................................................uaaaugcGuuaucugguauguaa...................    1        1    Fm3
......................................................................uaaaugcauuaCcugguauguaa...................    1        1    Fm3
......................................................................uaaaugcauuaucugUuauguaa...................    1        1    Fm3
......................................................................uaaaugcauuaucugguCuguaa...................    21       1    Fm3
......................................................................uaaauUcauuaucugguauguaa...................    2        1    Fm3
......................................................................uaaaugcauuGucugguauguaa...................    8        1    Fm3
......................................................................uaaaugcauuaucugguauguGa...................    1        1    Fm3
......................................................................uCaaugcauuaucugguauguaa...................    1        1    Fm3
......................................................................uaaaugcauuaucuggAauguaa...................    17       1    Fm3
......................................................................uaaaugcauuaucugguaAguaa...................    1        1    Fm3
......................................................................uaaaugcauuaucugguauAuaa...................    1        1    Fm3
......................................................................uaaaugNauuaucugguauguaa...................    1        1    Fm3
......................................................................uaaaugcauuaucAgguauguaa...................    2        1    Fm3
......................................................................uaaaugcauuUucugguauguaa...................    4        1    Fm3
......................................................................uaaaAgcauuaucugguauguaa...................    1        1    Fm3
......................................................................uaaaugUauuaucugguauguaa...................    1        1    Fm3
......................................................................uaaaugcauuaucugguGuguaa...................    22       1    Fm3
......................................................................uaaaugcauuaucugguauguaa...................    4866     0    Fm3
......................................................................uaaaugcauuaGcugguauguaa...................    2        1    Fm3
......................................................................uaaaugcauuaucugguauguaU...................    537      1    Fm3
......................................................................uaaaugcauuaucugguaGguaa...................    1        1    Fm3
......................................................................uaaaugcauuaucuggCauguaa...................    16       1    Fm3
......................................................................uaaaugcauuaucugguauguaC...................    1        1    Fm3
```

```
                                  Star                                Mature
cuagauguuguuuuguugauaccuguggaggguucguaucagaggugcauuugcagugaaguauucugugugaaaugcauuaucugguauguaacucucgcauguugugcagc
......................................................................uaaaugcauuaucugguaugCaa...................   1    1    Fm3
......................................................................uaaauAcauuaucugguauguaa...................   1    1    Fm3
......................................................................uaaaugcauuaucugguUuguaa...................   11   1    Fm3
......................................................................uaCaugcauuaucugguauguaa...................   1    1    Fm3
......................................................................Naaaugcauuaucugguauguaa...................   2    1    Fm3
......................................................................uaaaCgcauuaucugguauguaa...................   1    1    Fm3
......................................................................uaaaugcauuCucugguauguaa...................   6    1    Fm3
......................................................................uaaaugcauuaucGgguauguaa...................   1    1    Fm3
......................................................................uaaaugcauuaucugguaCguaa...................   1    1    Fm3
......................................................................uaaaugcauuaucugguauguUa...................   5    1    Fm3
......................................................................uaaaugcauuaucugguauguaac..................   28   0    Fm3
......................................................................uaaaugcauuaucugguauguaaU..................   51   1    Fm3
......................................................................uaaaugcauuaucugguauguaaA..................   27   1    Fm3
......................................................................uaaaugcauuaucugguauguaaAu.................   1    1    Fm3
......................................................................uaaaugcauuaucugguauguaaUu.................   3    1    Fm3
.......................................................................aaaugcauuaucugguau.......................   1    0    Fm3
.......................................................................aaaugcauuaucugguaug......................   12   0    Fm3
.......................................................................aaaugAauuaucugguaug......................   1    1    Fm3
.......................................................................aaaugcauuCucugguaugu.....................   1    1    Fm3
.......................................................................Uaaugcauuaucugguaugu.....................   7    1    Fm3
.......................................................................aaaugcauuauGugguaugu.....................   1    1    Fm3
.......................................................................aaaugcauuaucugguaugu.....................   33   0    Fm3
.......................................................................aaaugcauuaucugguUugua....................   1    1    Fm3
.......................................................................Uaaugcauuaucugguaugua....................   13   1    Fm3
.......................................................................aaaugcauuaGcugguaugua....................   2    1    Fm3
.......................................................................aaaugcauuaucugguaugua....................   71   0    Fm3
.......................................................................aaaugcauuaucugguauguaa...................   4    0    Fm3
.......................................................................Uaaugcauuaucugguauguaa...................   1    1    Fm3
........................................................................aaugcauuaucugguaug......................   5    0    Fm3
........................................................................aaugcauuaucugguaugu.....................   2    0    Fm3
........................................................................aaugcauuaucugguaCgu.....................   1    1    Fm3
........................................................................aaugcauuaucugguaugua....................   10   0    Fm3
........................................................................aaugcauuaucugguauguaa...................   6    0    Fm3
.........................................................................augcauuaucugguaugu.....................   13   0    Fm3
.........................................................................augcauuaucugguaugua....................   2    0    Fm3
..........................................................................ugcauuaucugguaugua....................   3    0    Fm3
```
